# Complete genome sequence of *Oligella urethralis* MSHR-50412PR, isolated from an ear discharge swab of a child with chronic suppurative otitis media

**DOI:** 10.1128/mra.01071-23

**Published:** 2024-01-26

**Authors:** Pappu K. Mandal, Alexander Cleanthous, Vanessa Rigas, Mariana Kleinecke, Katrina Lawrence, Amanda J. Leach, Heidi Smith-Vaughan, Peter S. Morris, Jemima Beissbarth, Robyn L. Marsh

**Affiliations:** 1 Child Health Division, Menzies School of Health Research, Charles Darwin University, Darwin, Northern Territory, Australia; 2 Global and Tropical Health Division, Menzies School of Health Research, Charles Darwin University, Darwin, Northern Territory, Australia; 3 Health and Human Science, Charles Darwin University, Darwin, Northern Territory, Australia; 4 School of Health Sciences, University of Tasmania, Launceston, Tasmania, Australia; Rochester Institute of Technology, Rochester, New York, USA

**Keywords:** *Oligella*, otitis media, CSOM, genome

## Abstract

*Oligella urethralis* are opportunistic pathogens typically associated with genitourinary infections. Here, we report the complete genome for an *Oligella urethralis* isolate recovered from ear discharge of a child with chronic suppurative otitis media (strain MSHR-50412PR). The genome comprises 2.58 Mb, with 2,448 coding sequences and 46.26% average GC content.

## ANNOUNCEMENT

*Oligella urethralis* can cause genitourinary infections and occasionally infect sterile body fluids (1). Microbiome charcterization of ear discharge swabs has detected *Oligella* operational taxonomic units (OTUs) among patients with chronic suppurative otitis media (CSOM) (2, 3). However, genome sequences for ear-associated *Oligella* spp. are lacking.

**Fig 1:**
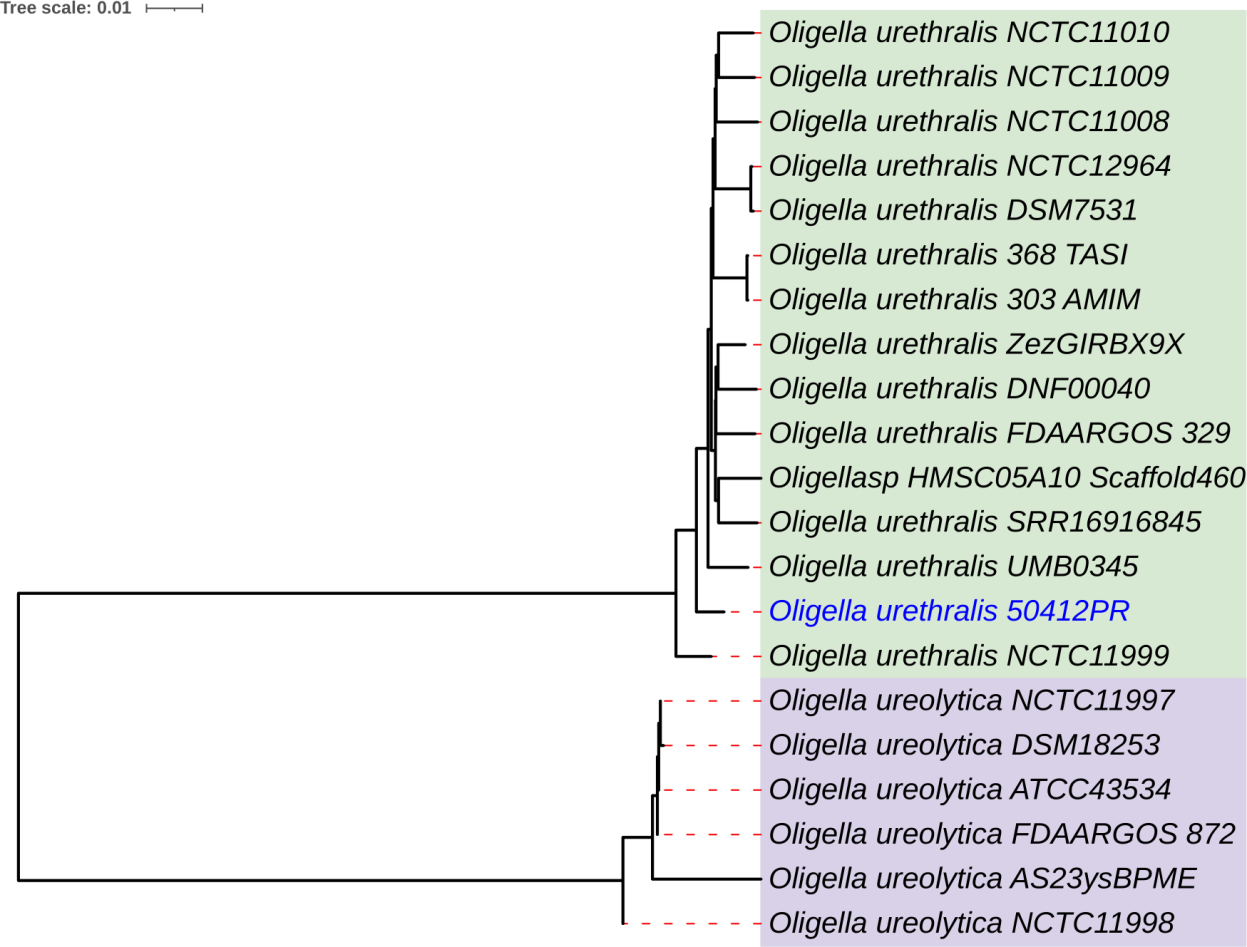
Whole genome sequence-based phylogenetic relationship of *Oligella urethralis MSHR-50412PR* to *Oligella* spp. genomes available in GenBank NCBI database. *Oligella* spp. genomes were downloaded from GenBank on 18 November 2022. The phylogeny was constructed using TYGS pipeline (12), and the image was generated using iTOL (13).

*O. urethralis* MSHR-50412PR was isolated from a baseline ear discharge swab collected in 2016 from a child residing in the Northern Territory, Australia, enrolled in a randomized controlled trial comparing CSOM treatments (4). The swab was stored in skim milk-tryptone-glucose-glycerin medium (5) at −80°C. Strain was isolated by inoculating 10 µL of the medium onto MacConkey without salt agar (ThermoFisher Scientific) and incubating at 37°C in 5% CO_2_ for 48 h. Colonies suggestive of *Oligella* spp. (1) were subcultured on Horse Blood Agar (ThermoFisher Scientific) with 24-h incubation at 37°C in 5% CO_2_, then identified using matrix-assisted laser desorption ionization-time of flight mass spectrometry (Bruker, USA). DNA was extracted from pure colonies (1/4 of a 10 µL loop) suspended in 360 µL of enzymatic lysis buffer (20 mM TrisHCl pH 8.0, 2 mM EDTA, 1.2% Triton, and 20 mg mL^−1^ lysozyme) using a QIAamp DNA Mini Kit bacterial protocol without modification (Qiagen, Australia) (6). DNA purity and quality were assessed using a Qubit double-stranded DNA high-sensitivity assay kit (ThermoFisher Scientific) and agarose gel electrophoresis. Sequencing was performed using Oxford Nanopore MinIONMk1C and Illumina Novaseq6000, using fresh DNA for each library. For Nanopore, a rapid barcoding kit (SQK-RB114.96) was used for library preparation; a flow cell priming kit (EXP-FLP004) and a FLO-MIN106(R 9.4) flow cell were used for sequencing. Data acquisition was done using MinKNOW v22.8.9, and Guppy v6.2.7 (Oxford Nanopore) was used for base-calling and demultiplexing, generating 220,893 reads (N50:13404). Quality filtering and trimming were done using NanoFilt v2.8.0 (7). For Illumina, 150-bp paired-end reads were sequenced by a commercial provider (AGRF, Australia) using Illumina DNA prep PCR-Free library preparation workflow (v1.5 chemistry, XP-lane splitter kit), generating 7,816,232 reads. Quality filtering and trimming were done using Trimmomatic v0.36 (8). *De novo* hybrid assembly of Illumina and Nanopore-filtered reads was done using Unicycler v0.5.0 (9), generating a single circular contig of 25,80,519 bp (N50:2580519) with 368× Illumina reads and 473× Nanopore reads coverage. BLASTn pairwise assembly comparison was performed against the *O. urethralis* strain FDAARGOS_329 reference genome (GenBank accession: CP027417) (10) using ACT v18.2.0 (11), and the origin of the assembly was selected to match the reference genome. Whole genome-based taxonomy was determined using TYGS pipeline (12) with reference to all *Oligella* spp. genomes available in GenBank on 18/11/22. The phylogenetic tree (Fig. 1) was generated using iTOL (13). The genome was annotated using Prokka v1.14.6 (14). Virulence and antibiotic resistance genes were identified using VFanalyzer (15) and Staramr v0.9.1 (16), respectively. Default parameters were used for all software.

The complete genome includes 2,448 coding sequences and 46.26% average GC content. VFanalyzer identified 59 virulence genes, including genes encoding capsule, endotoxin, enolase, siderophore acinetobactin, and a type VI secretion system. Staramr identified one antibiotic resistance gene encoding a β-lactamase (*blaCARB-8*). In phenotypic antimicrobial susceptibility tests done using the calibrated dichotomous sensitivity (CDS) method (17), MSHR-50412PR demonstrated resistance to penicillin, ampicillin, and oxacillin and susceptibility to amoxycillin-clavulanic acid, cefoxitin, ceftazidime, ciprofloxacin, gentamicin, erythromycin, and cotrimoxazole.

## Data Availability

The complete genome of *Oligella urethralis* MSHR-50412PR and all the raw reads have been deposited in GenBank under the accession numbers CP137240 and PRJNA1020600. The version described in this paper is the first version.
